# Lithium nephropathy in Bipolar Disorder: a clinical challenge

**DOI:** 10.1192/j.eurpsy.2024.890

**Published:** 2024-08-27

**Authors:** C. Alario-Ruiz, B. Arribas-Simon, P. Martinez.Gimeno, M. Calvo-Valcarcel, O. Martin-Santiago

**Affiliations:** Hospital Clinico Universitario, Valladolid, Spain

## Abstract

**Introduction:**

Lithium nephropathy can occur in long-term lithium-treated bipolar disorder patients. Key risk factors include duration of lithium exposure, cumulative dose, acute intoxication episodes, advanced age, and comorbidities such as hypertension, diabetes mellitus, hyperparathyroidism, and hyperuricemia, along with concurrent use of antipsychotics. The clinical presentation is gradual, with mild proteinuria, often accompanied by arginine vasopressin resistance. Histological studies show a correlation between interstitial fibrosis and cumulative lithium duration. Approximately 15 to 25 per cent of exposed patients may experience a gradual decline in glomerular filtration rate. The outcome after lithium discontinuation varies.

**Objectives:**

This case study aims to analyze and document the clinical presentation, diagnosis, and management of lithium nephropathy in a patient with Bipolar Disorder.

**Methods:**

We gathered data on the medical history, lab results, and treatment approach for a patient with Bipolar Disorder.

**Results:**

The patient, a 50-year-old woman, had been under the care of Psychiatry since 2008 due to a diagnosis of Bipolar Disorder Type I. During this time, she had experienced depressive and manic episodes but had not presented significant symptom decompensation for the past 14 years, successfully managed with lithium at a current dose of 600 mg per day. However, on this occasion, the patient sought hospitalization due to recent behavioural disturbances, including restlessness, disinhibition, abrupt changes in behaviour, pressured speech, sleep problems, agitation, and aggression. The patient also reported an increased sense of polyuria and polydipsia. Evaluation in the emergency department revealed elevated lithium levels of 1.47 mmol/L and hypokalemia, that justified lithium withdrawal. After lithium levels decreased, an estimated glomerular filtration rate remained low. She was diagnosed with lithium nephropathy, an adverse effect of long-term lithium therapy. Treatment with lithium changed to sodium valproate. Treatment with asenapine started and sustained for two months. Over the following two years, the patient experienced four additional hospital admissions in Psychiatry due to manic episodes.

**Conclusions:**

Long-term lithium therapy can lead to lithium nephropathy with symptoms such as polyuria, polydipsia, and acute kidney failure. Consistent monitoring of patients receiving lithium is crucial to detect potential adverse effects.This case highlights the challenges in managing bipolar patients, as discontinuing lithium exacerbated symptoms despite switching to sodium valproate for nephropathy prevention. Long-term lithium treatment, while effective for bipolar disorder, poses significant renal risks. We emphasize continuous renal function monitoring and assessing the risk-benefit of lithium treatment while actively researching lithium nephropathy and its impact on glomerular function.

**Disclosure of Interest:**

None Declared

